# Discussion on the mechanism of Bao-Yuan Yang-Xin Decoction in treating coronary heart disease based on network pharmacology and molecular docking

**DOI:** 10.1097/MD.0000000000042882

**Published:** 2025-07-04

**Authors:** Chuanqian Liu, Zhenzhen Liu, Xueting Zhang, Xipeng Yan

**Affiliations:** a Department of Traditional Chinese Medicine, Jining No. 1 People’s Hospital, Jining, Shandong Province, China.

**Keywords:** Bao-Yuan Yang-Xin Decoction, coronary heart disease, molecular docking, network pharmacology

## Abstract

This study aimed to explore the potential therapeutic mechanisms of the Bao-Yuan Yang-Xin Decoction (BYXD) in the treatment of coronary heart disease (CHD) by integrating network pharmacology with the specificity of molecular docking methods. Effective components and their potential targets of BYXD were screened from the Traditional Chinese Medicine Systems Pharmacology and BATMAN-Traditional Chinese Medicine databases. Targets related to CHD were confirmed through the GeneCards database. A “drug–component–target” network was created using Cytoscape, and a protein–protein interaction network was established with STRING. Finally, biological enrichment analysis of the targets was performed using the Database for Annotation, Visualization and Integrated Discovery, and the top 20 highly enriched Kyoto Encyclopedia of Genes and Genomes pathways were further investigated. The pivotal active constituents of BYXD are identified as stigmasterol, quercetin, β-sitosterol, luteolin, and baicalein in this study. Major targets include PTGS2, PTGS1, NCOA2, ADRB2, SCN5A, RXRA, NCOA1, ESR1, AR, and DPP4. BYXD conveys its healing properties to CHD by controlling key communication pathways, including advanced glycation end products-receptor, tumor necrosis factor, HIF-1, and IL-17. Molecular docking shows a high degree of binding affinity between stigmasterol and PTGS2, while β-sitosterol demonstrates a notable affinity for NCOA1. The probable mechanisms underlying BYXD’s efficacy in CHD treatment are disclosed by applying network pharmacology and molecular docking. The integral components of BYXD are identified to selectively engage with a range of critical targets, modulating the associated signaling pathways that are pivotal in the pathophysiology of CHD. Such targeted modulation provides a substantive scientific basis for the clinical application of BYXD.

## 1. Introduction

Coronary artery disease, which is synonymous with coronary heart disease (CHD), persists as a major source of fatalities on a worldwide scale.^[1]^ CHD, a complex condition with multiple determinants, is shaped by a spectrum of influences including increased levels of low-density lipoprotein cholesterol, elevated triglycerides, diminished high-density lipoprotein cholesterol, inflammatory biomarkers including C-reactive protein, alongside hereditary, and behavioral elements. The core pathological process involves atherosclerotic plaques narrowing or obstructing the coronary arteries, triggering a decrease in blood and oxygen availability to the heart.^[2]^ The elderly population is at an increased risk factor because of increased vascular endothelial thickening, endothelial dysfunction, and thrombosis.^[3]^ Aging populations, notably in China, face escalating rates of cardiovascular disease incidence and mortality.^[4]^

Treatment protocols for CHD typically involve medications to regulate lipid levels, blood glucose, blood pressure, and inflammation, as well as antiplatelet aggregation and anti-atherosclerosis therapies. However, the long-term intake of these drugs could produce harmful secondary effects. Traditional Chinese Medicine (TCM), celebrated for its limited adverse reactions, has garnered widespread attention in the treatment of CHD.^[5,6]^ Recently, the integration of traditional and modern medicine has experienced notable improvements in the standardized diagnosis and treatment of cardiovascular diseases, with 22 industry guidelines and expert consensuses published specifically targeting the prevention and therapeutic approaches for CHD in the past decade.^[7]^ TCM in Cardiovascular Drug Discovery,^[8]^ a virtual special issue, presented 18 studies underscoring the therapeutic potential of TCM in both basic and clinical research. Their medical examinations have confirmed the successful outcomes of TCM in CHD therapy.

Recent advances in computational methods have revolutionized research approaches in TCM, particularly through the application of network pharmacology. This emerging field integrates systems biology, bioinformatics, and traditional pharmacology to decipher the complex mechanisms of multi-component, multi-target herbal formulations. Zhai et al utilized network pharmacology and molecular docking techniques to investigate the immunomodulatory mechanism of rhubarb peony decoction for treating ulcerative colitis and irritable bowel syndrome, identifying key active compounds and molecular targets involved in the therapeutic effects of this traditional formula.^[9]^ Zhang et al explored the transition towards artificial intelligence-based precision TCM through network pharmacology. Their research highlighted how computational models can predict drug–target interactions and optimize personalized treatment strategies based on individual patient characteristics.^[10]^ Zhou et al combined computational systems pharmacology, molecular docking, and experimental validation to investigate the protective mechanism of Li-Da-Qian mixture in treating glomerulonephritis. Their multi-layered approach revealed that this traditional formula modulates inflammatory pathways and immune responses through multiple active compounds and targets.^[11]^

Network pharmacology provides a structured method for gaining insight into the functioning of which TCM treats ischemic heart diseases.^[12]^ Moreover, an extensive systematic review coupled with meta-analysis showed the excellence of TCM in regulating glucose and lipid metabolism, hinting at a protective influence on the heart in diabetic patients with CHD.^[13]^ These findings collectively provide strong evidence for the effectiveness of TCM in treating CHD and create the prerequisites for further research dedicated to standardizing treatments and illuminating foundational principles.

In TCM, CHD belongs to the divisions of “chest obstruction and heart pain” and “true heart pain.” It is often treated by addressing “qi-yin deficiency” and “blood stasis,” focusing on revitalizing energy and resolving blood flow disruptions.^[14]^ After years of clinical practice, he developed the Bao-Yuan Yang-Xin Decoction (BYXD), which comprises *Astragalus membranaceus* (Huangqi), *Codonopsis pilosula* (Dangshen), *Ophiopogon japonicus* (Maidong), *Schisandra chinensis* (Wuweizi), *Ligusticum chuanxiong* (Chuanxiong), *Paeonia lactiflora* (Chic Shao), *Salvia miltiorrhiza* (Danshen), *Polygonatum sibiricum* (Huangjing), *Santalum album* (Tanalux), *Amomi fruticosus* (Sharen), and *Cinnamomum cassia* (Guizhi).^[15]^ Numerous studies have indicated the advantageous curative effects of BYXD in the treatment of CHD. Yan et al^[15]^ revealed productive healing strategies of BYXD in a patient with triple-vessel disease. Zhang et al^[16]^ discovered that BYXD is notably effective in thwarting the occurrence of restenosis post-stent by involving 30 patients who underwent coronary artery stenting. Additionally, Yan et al^[17]^ employed BYXD along with extra components to counteract the re-narrowing of the coronary arteries post-stent procedure. With an efficacy rate of 86%, the treatment group demonstrated superior outcomes compared to the control group’s 57%, accompanied by a lower incidence of adverse events. In essence, BYXD has been validated as a potent supplementary treatment within the extensive management of CHD.

This study employed a network pharmacology approach coupled with molecular docking method, to map out the interactions of BYXD’s active substances with the CHD-related biological targets. Network pharmacology offers a comprehensive view of the intricate web of interactions among various constituents and targets in herbal remedies, whereas molecular docking enables an exact forecast of the engagement between pharmaceutical agents and their prospective targets. The fusion of these 2 methodologies enables a deeper exploration of the molecular mechanisms and biological pathways entailed in the therapeutic success of BYXD in the treatment of CHD. Following the method, it consequently provides a more precise insight into its healing processes.

## 2. Materials and methods

### 2.1. Collection and screening of active ingredients and target points of BYXD

The active ingredients and target points of BYXD were gathered and filtered using the Traditional Chinese Medicine Systems Pharmacology (TCMSP) platform (https://old.tcmsp-e.com/tcmsp.php), with components having an oral bioavailability (OB) of at least 30% and a drug-likeness score of at least 0.18 selected as the criteria. As *O japonicus* (Maidong) was not available in the TCMSP database, the BATMAN-TCM database (http://bionet.ncpsb.org/batman-tcm/) was used to identify its active ingredients, which were subsequently screened based on the same OB and drug-likeness thresholds within the TCMSP database.

### 2.2. CHD-related gene targets and intersection targets of BYXD

CHD-related genes were retrieved via the GeneCards database (https://www.genecards.org/) using the keyword “coronary heart disease.” The intersection targets between BYXD components and CHD-related genes were identified and visualized using a Venn diagram created with the microbioinformatics platform (www.bioinformatics.com.cn). Potential therapeutic targets of BYXD for CHD were determined based on these intersections.

### 2.3. Construction of the BYXD–active ingredient–intersection target network

The “BYXD–active ingredient–intersection target” network was constructed and analyzed utilizing the Cytoscape 3.10.0 software. The key components and core targets of BYXD for treating CHD were identified through topological analysis of the network using the Network Analyzer tool integrated into Cytoscape.

### 2.4. Construction of protein–protein interaction (PPI) network

The intersection targets of BYXD for CHD treatment were integrated into the STRING database (https://string-db.org/) at a confidence level surpassing 0.9. The species was specified as “*Homo sapiens*,” and a PPI network was constructed.

### 2.5. Gene Ontology (GO) functional and Kyoto Encyclopedia of Genes and Genomes (KEGG) pathway enrichment analysis

Biological process (BP), cellular component (CC), and molecular function (MF) analyses were operated on the Database for Annotation, Visualization, and Integrated Discovery platform (https://david.ncifcrf.gov/). Functional categories with significant enrichment (false discovery rate [FDR] < 0.05) were selected, and the top 20 KEGG pathways highly enriched in BYXD treatment of CHD were analyzed to unveil the essential pathways in action.

### 2.6. Molecular docking verification

Molecular docking verification was conducted using core genes and important small molecule ligands. Mol2 format files were downloaded from the TCMSP website. The protein names corresponding to the core genes were translated using UniProt (https://www.uniprot.org/), and their 3D structures were retrieved from the Protein Data Bank (PDB; http://www.rcsb.org/) and saved in PDB format. Water molecules and small molecule ligands were eliminated from the protein 3D structures using PyMOL software, leaving only the receptor structure. Hydrogens were added and charges were computed using Autodock Tools software. The processed protein receptor and small molecule ligand files were stored in pdbqt format. Grid parameters and coordinates for the protein receptors were set using Grid Box, and the binding energies of the small molecule ligands and protein receptors were calculated. A heatmap of molecular docking binding energies was generated using the microbioinformatics platform. Finally, by applying PyMOL software, the molecular docking conformations were visualized and illustrated.

### 2.7. Ethics approval and consent to participate

This study was approved by the Institutional Review Board of Jining No. 1 People’s Hospital and was conducted in accordance with the Declaration of Helsinki. This study used the data from GeneCards database, and the informed consent for this study was waived by the review board.

## 3. Results

### 3.1. Active compounds and their target points of BYXD

In total, there were 193 active compounds sorted out of BYXD using the TCMSP and BATMAN-TCM databases. These included 20 compounds from *A membranaceus* (Huangqi), 21 from *C pilosula* (Dangshen), 18 from *O japonicus* (Maidong), 8 from *S chinensis* (Wuweizi), 7 from *L chuanxiong* (Chuanxiong), 29 from *P lactiflora* (Chishao), 65 from *S miltiorrhiza* (Danshen), 12 from *P sibiricum* (Huangjing), 3 from *S album* (Tanxiang), 10 from *Amomum villosum* (Sharen), and 7 from *C cassia* (Guizhi). Table 1 lists the top 20 compounds based on their OB values. The number of target points for each compound were as follows: 455 for Huangqi, 198 for Dangshen, 283 for Maidong, 28 for Wuweizi, 38 for Chuanxiong, 154 for Chishao, 892 for Danshen, 146 for Huangjing, 97 for Tanxiang, 75 for Sharen, and 69 for Guizhi. These target points were converted into corresponding gene names using UniProt. Following the consolidation and elimination of duplicates, a total of 489 unique target points were obtained.

**Table 1:** Major active compounds of Bao-Yuan Yang-Xin Decoction (ranked by OB value, top 20).

Mol ID	Active compound name	OB (%)	DL	Source
MOL007064	Przewalskin b	110.32	0.44	*Salvia miltiorrhiza* (Dan Shen)
MOL000398	Isoflavanone	109.99	0.3	*Astragalus membranaceus* (Huang Qi)
MOL007132	(2R)-3-(3,4-dihydroxyphenyl)-2-[(Z)-3-(3,4-dihydroxyphenyl)acryloyl]oxy-propionic acid	109.38	0.35	*Salvia miltiorrhiza* (Dan Shen)
MOL007140	(Z)-3-[2-[(E)-2-(3,4-dihydroxyphenyl)vinyl]-3,4-dihydroxy-phenyl]acrylic acid	88.54	0.26	*Salvia miltiorrhiza* (Dan Shen)
MOL001918	Paeoniflorgenone	87.59	0.37	*Paeonia lactiflora* (Chic Shao)
MOL008647	N-Trans-Feruloyltyramine	86.71	0.26	*Ophiopogon japonicus* (Mai Dong)
MOL000546	Diosgenin	80.88	0.81	*Polygonatum sibiricum* (Huang Jing)
MOL007150	(6S)-6-hydroxy-1-methyl-6-methylol-8,9-dihydro-7H-naphtho[8,7-g]benzofuran-10,11-quinone	75.39	0.46	*Salvia miltiorrhiza* (Dan Shen)
MOL000378	7-O-methylisomucronulatol	74.69	0.3	*Astragalus membranaceus* (Huang Qi)
MOL007058	Formyltanshinone	73.44	0.42	*Salvia miltiorrhiza* (Dan Shen)
MOL004941	(2R)-7-hydroxy-2-(4-hydroxyphenyl)chroman-4-one	71.12	0.18	*Polygonatum sibiricum* (Huang Jing)
MOL007120	Miltionone Ⅱ	71.03	0.44	*Salvia miltiorrhiza* (Dan Shen)
MOL000392	Formononetin	69.67	0.21	*Astragalus membranaceus* (Huang Qi)
MOL000433	FA	68.96	0.71	*Astragalus membranaceus* (Huang Qi), *Ligusticum chuanxiong* (Chuan Xiong)
MOL007105	Epidanshenspiroketallactone	68.27	0.31	*Salvia miltiorrhiza* (Dan Shen)
MOL001925	Paeoniflorin_qt	68.18	0.4	*Paeonia lactiflora* (Chic Shao)
MOL000438	(3R)-3-(2-hydroxy-3,4-dimethoxyphenyl)chroman-7-ol	67.67	0.26	*Astragalus membranaceus* (Huang Qi)
MOL002140	Perlolyrine	65.95	0.27	*Codonopsis pilosula* (Dang Shen), *Ligusticum chuanxiong* (Chuan Xiong)
MOL005321	Frutinone A	65.9	0.34	*Codonopsis pilosula* (Dang Shen)
MOL007016	Paeoniflorigenone	65.33	0.37	*Paeonia lactiflora* (Chic Shao)

DL = drug-likeness, OB = oral bioavailability.

### 3.2. Intersection of targets between BYXD and CHD

**Figure 1.**
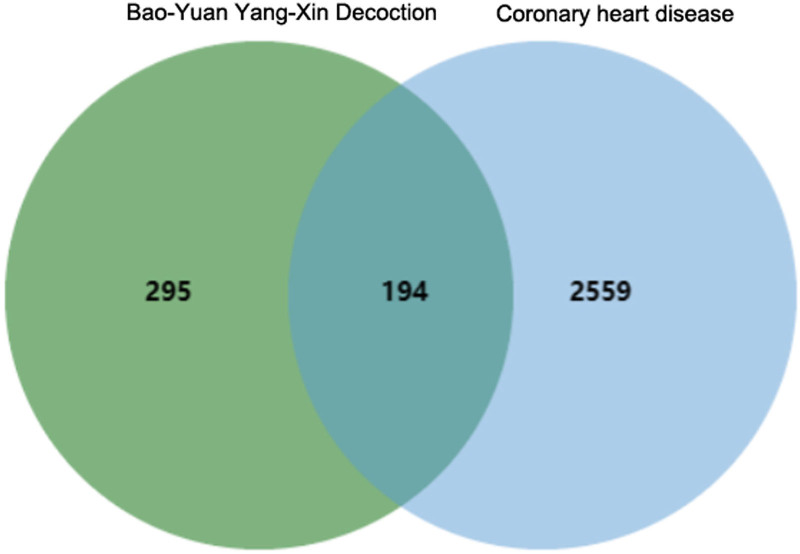
Venn diagram showing the intersection of targets between Bao-Yuan Yang-Xin Decoction and coronary heart disease. One hundred ninety-four targets of active components from Bao-yuan Yang-xin Tang (left), 2753 coronary heart disease-related targets (right), and 194 intersection targets shared between them (middle).

CHD-related genes were searched in the GeneCards database, resulting in 2559 disease targets closely associated with CHD. The overlap within the active compound targets of BYXD and the CHD disease targets was identified using bioinformatics tools, and the results were visualized in a Venn diagram. This analysis revealed an intersection of 194 targets between BYXD and CHD, as shown in Figure 1.

### 3.3. Construction of the “BYXD–active ingredients–intersection targets” network

**Figure 2.**
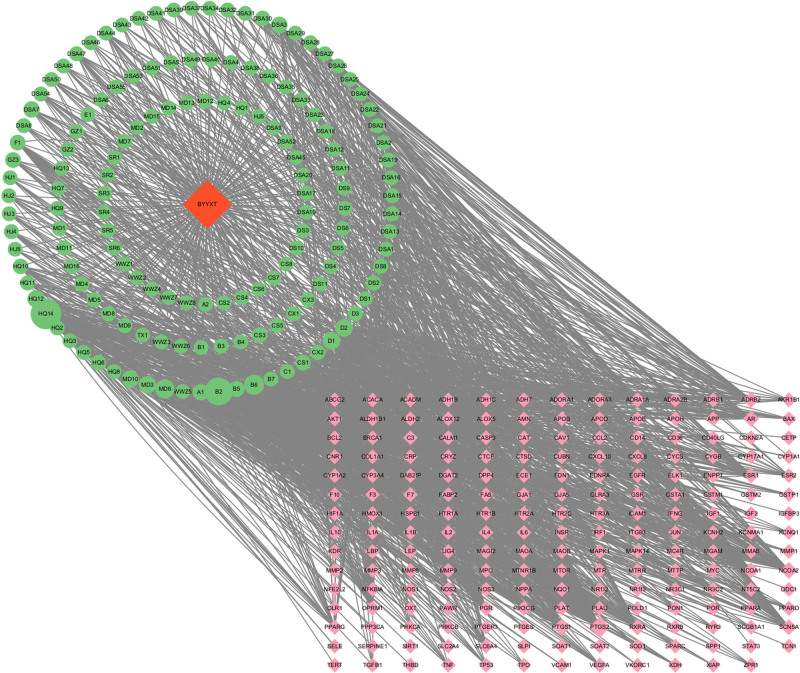
Network depicting the intersection of targets between Bao-Yuan Yang-Xin Decoction, its active ingredients, and coronary heart disease genes. Green circular nodes represent drug chemical components, red square nodes represent the intersection targets between Bao-Yuan Yang-Xin Decoction and coronary heart disease genes. The strength of the association between gene targets and drug components is indicated by the size of the green nodes, with larger nodes indicating a stronger association. Interactions are represented by connecting lines.

The “BYXD–active ingredients–intersection targets” network was generated by using Cytoscape version 3.10.0 (Fig. 2). In this network, green circular nodes represent the chemical constituents of BYXD, while red square nodes denote the intersection targets between BYXD and CHD genes. The degree of association between the gene targets and the chemical constituents is indicated by the size of the green nodes, with larger nodes representing stronger associations. Interactions between these 2 entities are represented by connecting lines.

The network topology parameters of BYXD components treating CHD were analyzed using the Network Analyzer tool within Cytoscape 3.10.0. The results, ranked by degree values, illustrated that stigmasterol held a degree of 184, betweenness centrality of 20,918.521, and closeness centrality of 0.43422732. Stigmasterol is predicted to be the primary constituent of BYXD in treating CHD. Quercetin, beta-sitosterol, luteolin, and baicalein are also considered major effective components of BYXD in treating CHD. Detailed information is presented in Table 2. In the network, PTGS2 had a degree of 101.0, betweenness centrality of 9972.386, and closeness centrality of 0.47486034. PTGS2 is predicted to be the most critical target for BYXD in treating CHD. PTGS1, NCOA2, ADRB2, SCN5A, RXRA, NCOA1, ESR1, AR, and DPP4 are also relatively important targets. See Table 3 for details.In the network, PTGS2 had a degree of 101.0, betweenness centrality of 9972.386, and closeness centrality of 0.47486034. PTGS2 is predicted to be the most critical target for BYXD in treating CHD. PTGS1, NCOA2, ADRB2, SCN5A, RXRA, NCOA1, ESR1, AR, and DPP4 are also relatively important targets. See Table 3 for details.

**Table 2:** Major effective components of Bao-Yuan Yang-Xin Decoction in treating coronary heart disease.

Components	Degree	Betweenness	Closeness
Stigmasterol	184	20918.521	0.434227
Quercetin	95	25941.547	0.493469
Beta-sitosterol	80	4337.2344	0.407186
Luteolin	77	8338.124	0.427673
Baicalein	45	4560.8667	0.410133
Isorhamnetin	42	2322.399	0.408163
Kaempferol	39	4177.9204	0.425532
Methyl icosa-11,14-dienoate	37	2147.5933	0.42236
7-O-methylisomucronulatol	24	1408.267	0.411125
Dopamine D1 receptor	24	3043.7688	0.411125
Diosgenin	24	1534.1405	0.39953
Formononetin	21	1094.6459	0.407186

**Table 3:** Major targets of Bao-Yuan Yang-Xin Decoction in treating coronary heart disease.

Gene	Degree	Betweenness	Closeness
PTGS2	101	9972.386	0.47486
PTGS1	63	4125.708	0.429293
NCOA2	60	3729.685	0.410628
ADRB2	50	2304.643	0.411622
SCN5A	48	2089.454	0.405728
RXRA	48	2147.137	0.409639
NCOA1	46	1698.597	0.372807
ESR1	43	1173.238	0.349076
AR	41	1571.907	0.382022
DPP4	40	1584.294	0.380313
PGR	40	1768.85	0.354906
ADRA1A	36	924.1705	0.362473

### 3.4. Construction of the PPI network of BYXD in CHD

**Figure 3.**
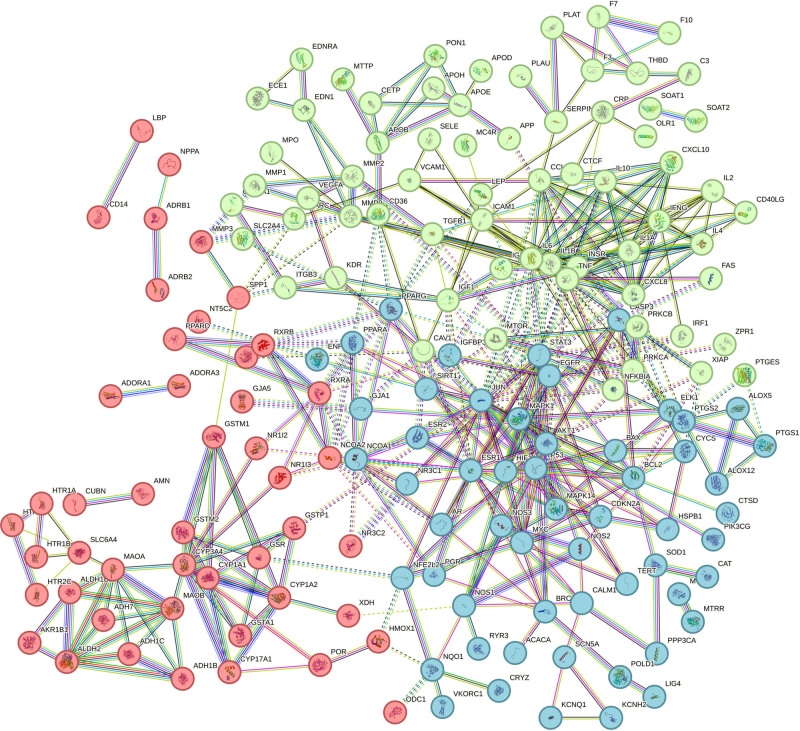
The PPI network of BYXD in CHD. One hundred ninety-four nodes and 462 edges, representing 194 interacting protein genes, and 462 interaction connections. (This is repeated in the original text). BYXD = Bao-Yuan Yang-Xin Decoction, CHD = coronary heart disease, PPI = protein–protein interaction.

The intersection targets of BYXD in treating CHD were fed into the STRING platform for creating a PPI network, as shown in Figure 3. The expected number of edges was 101. The assembled PPI network was composed of 194 nodes and 462 edges, representing 194 interacting protein genes and 462 interactions, respectively. The *P*-value of PPI enrichment was < 1.0e-16.

### 3.5. GO and KEGG analysis

**Figure 4.**
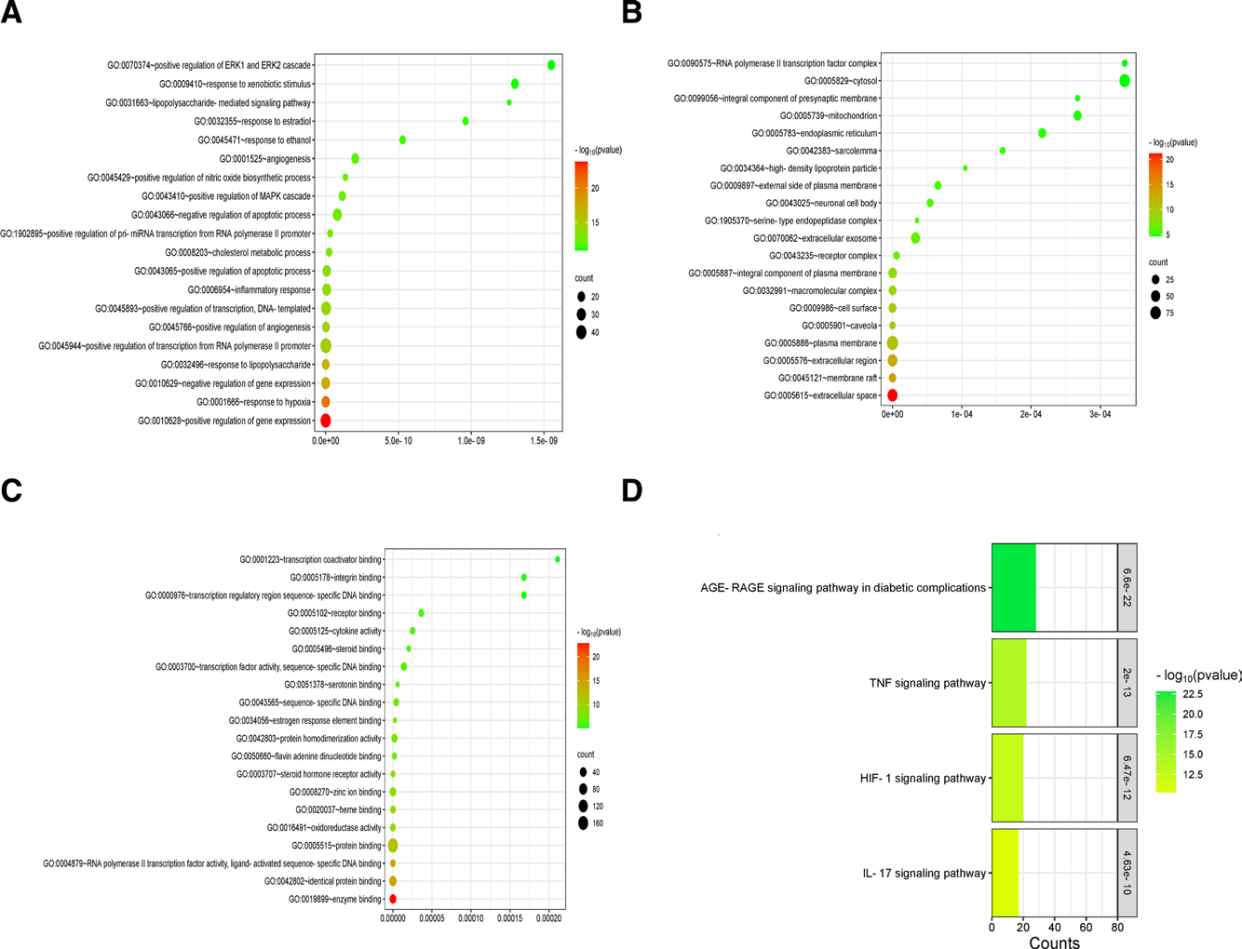
Gene Ontology biological process enrichment analysis and significantly enriched signaling pathways. (A) Biological processes (Y-axis), FDR values (X-axis), and *P*-values indicated by color variation, with green representing higher values and red representing lower values. The bubble size corresponds to the number of genes; (B) molecular functions (Y-axis), FDR values (X-axis), and *P*-values indicated by color variation, with green representing higher values and red representing lower values. The bubble size corresponds to the number of genes; (C) cellular components (Y-axis), FDR values (X-axis), and *P*-values indicated by color variation, with green representing higher values and red representing lower values. The bubble size corresponds to the number of genes; (D) signaling pathways (Y-axis), gene counts (X-axis), and *P*-values shown by color variation, with green representing lower values and red representing higher values. FDR = false discovery rate.

The 194 target genes obtained from the PPI network were subjected to GO and KEGG pathway analysis by relying on the Database for Annotation, Visualization, and Integrated Discovery. The GO enrichment process was initiated from 3 distinct biological perspectives: BP, CC, and MF. GO enrichment entries with a FDR <0.05 were selected, totaling 1115 entries, including 867 BP, 98 CC, and 150 MFs. The top 20 enriched GO terms were visualized using the Microbial Information Platform and presented as bubble charts, as shown in Figure 4. The analysis of these enriched GO terms revealed that the genes were predominantly participating in BP such as positive regulation of gene expression, response to hypoxia, negative regulation of gene expression, response to lipopolysaccharide, and positive regulation of transcription from RNA polymerase II promoter. In terms of CC, they mainly included the extracellular space, membrane rafts, extracellular regions, plasma membranes, and cell surfaces. For MFs, the focus was on enzyme binding, identical protein binding, RNA polymerase II transcription factor activity (ligand-activated sequence-specific DNA binding), protein binding, and oxidoreductase activity.

The KEGG pathway analysis, as depicted in the Figure 4, reveals the top 20 significantly enriched pathways (FDR < 0.05). These pathways include the advanced glycation end products-receptor (AGE-RAGE) signaling pathway in diabetic complications, the tumor necrosis factor (TNF) signaling pathway, the hypoxia-inducible factor 1 (HIF-1) signaling pathway, and the interleukin-17 (IL-17) signaling pathway.

### 3.6. Molecular docking validation

**Figure 5.**
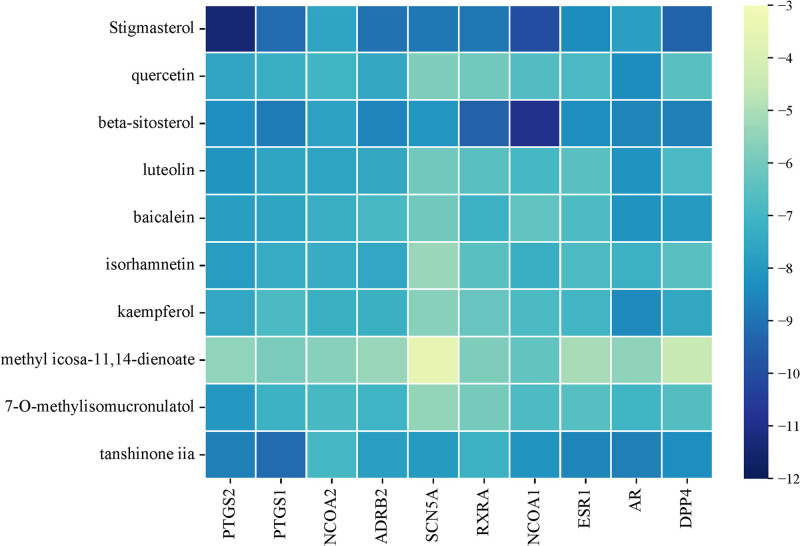
Heatmap of molecular docking binding energies. Active compounds (Y-axis), disease targets (X-axis), and molecular binding energy (indicated by color variation, with darker colors representing lower values and lighter colors representing higher values).

**Figure 6.**
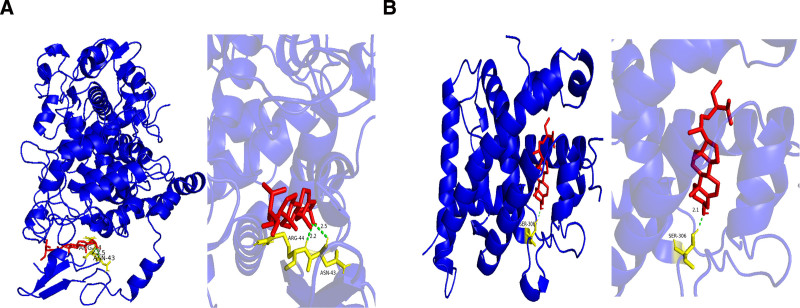
Molecular docking analysis of plant sterols with target proteins. (A) The full protein structures (blue ribbon) with docked sterol ligands, displaying the overall binding context of stigmasterol with PTGS2 (left) and β-sitosterol with NCOA1 (right); (B) zoomed-in views of the binding pockets, highlighting specific molecular interactions between the sterol compounds (red/yellow) and their respective protein targets within the active sites.

Molecular docking analysis was calculated using AutoDock software for the 10 key active components screened from BYXD and their interaction with 10 core targets. A heatmap was generated using the microinformatics platform to visualize the results (see Table 4 for the numerical data, and Fig. 5 for the heatmap). The analysis showed that out of 100 active component–target interactions, 98 demonstrated binding energies that were inferior to −5 kcal/mol, suggesting strong binding potential between the active components and the core targets. Additionally, 2 interactions with particularly low binding energies (< −10 kcal/mol) were selected for visualization using PyMOL software: the binding of stigmasterol to PTGS2 and β-sitosterol to NCOA1. The corresponding molecular docking conformation diagrams are presented in Figure 6, respectively.

**Table 4:** Molecular docking binding energy results.

Components	binding energy(kcal/mol)									
	PTGS2	PTGS1	NCOA2	ADRB2	SCN5A	RXRA	NCOA1	ESR1	AR	DPP4
Stigmasterol	-11.37	-9.16	-7.64	-9.01	-8.87	-8.86	-9.99	-8.31	-7.77	-9.34
Quercetin	-7.54	-7.26	-7.02	-7.47	-5.79	-6.05	-6.69	-6.81	-8.31	-6.55
Beta-sitosterol	-8.29	-8.79	-7.71	-8.49	-8.05	-9.39	-10.92	-8.29	-8.54	-8.66
Luteolin	-8.11	-7.63	-7.67	-7.47	-5.99	-6.49	-6.91	-6.51	-8.16	-6.82
Baicalein	-7.81	-7.61	-7.27	-6.87	-6.02	-7.14	-6.36	-6.73	-8.15	-7.95
Isorhamnetin	-7.86	-7.33	-7.35	-7.53	-5.32	-6.53	-7.28	-6.76	-7.16	-6.51
Kaempferol	-7.51	-6.79	-7.19	-7.21	-5.63	-6.25	-6.77	-6.98	-8.42	-7.50
Methyl icosa-11,14-dienoate	-5.48	-5.85	-5.61	-5.35	-3.56	-5.78	-6.32	-5.04	-5.52	-4.49
7-O-methylisomucronulatol	-8.00	-7.12	-6.88	-7.06	-5.41	-5.93	-6.73	-6.56	-7.03	-6.65
Tanshinone iia	-8.66	-9.16	-6.91	-7.75	-7.99	-7.15	-8.12	-8.52	-8.63	-8.30

## 4. Discussion

CHD is categorized in TCM as “chest obstruction with heart pain.” The BYXD is a traditional herbal formula that works through mechanisms of qi tonification, yin nourishment, essence generation, and blood circulation enhancement, making it suitable for CHD patients with a combination of Qi and Yin insufficiency coupled with blood stagnation. To further explore BYXD mechanisms in CHD, the network pharmacology and molecular docking techniques were utilized to clarify the interaction network between herbal components and biological targets, providing scientific evidence for clinical application.

This study identified the primary chemical constituents of BYXD for treating CHD as stigmasterol, quercetin, β-sitosterol, luteolin, and baicalin. Stigmasterol and β-sitosterol possess potent antioxidant and cholesterol-lowering effects,^[18–20]^ which help to reduce the severity of atherosclerosis. Furthermore, β-sitosterol has protective effects on hypoxic/reoxygenated and ischemic/reperfused myocardial cells.^[21]^ Quercetin and luteolin are flavonoids; research has shown^[22]^ that flavonoids can reduce the generation of reactive oxygen species, exerting antioxidant effects that protect the cardiovascular system. Moreover, flavonoids impede platelet incitement and coagulation.^[23]^ Kangmei Wen et al^[24]^ sumarazied that quercetin supports heart health by improving function, preventing muscle enlargement, reducing clot risk, widening vessels, and managing lipid levels. Li et al^[25]^ found that quercetin exerts its beneficial effects on coronary artery atherosclerosis primarily by inhibiting the Gal-3-NLRP3 signaling pathway, which in turn helps to curb inflammation and oxidative stress. Additionally, quercetin amplifies the body’s innate energy production, thereby alleviating the damage caused by myocardial hypoxia.^[26]^ Ding et al^[27]^ demonstrated that luteolin can alleviate inflammatory responses, decrease macrophage accumulation and lipid deposition, thereby improving coronary artery atherosclerosis. Baicalin hinders the growth rate of vascular smooth muscle cells by regulating downstream target genes, such as long non-coding RNAs, which delays the development of atherosclerotic plaques.^[28]^ Moreover, baicalin has a protective effect against ischemia/reperfusion injury in myocardial cells.^[29]^ In conclusion, the synergistic effects of these key active ingredients in BYXD, working through various regulatory mechanisms including lipid regulation, antioxidant activity, anti-inflammatory effects, and enhanced circulation, comprehensively improve cardiovascular function in CHD patients.

Network analysis using Cytoscape has identified PTGS2, PTGS1, NCOA2, ADRB2, SCN5A, RXRA, NCOA1, ESR1, AR, and DPP4 as key targets for the treatment of CHD with BYXD. PTGS1 and PTGS2 play crucial roles in prostaglandin biosynthesis and contribute to a spectrum of normal and pathological mechanis, including inflammation, pain, fever, and vasodilation/vasoconstriction. Overexpression of PTGS2 is associated with multiple inflammatory diseases,^[30,31]^ and considering inflammation as a key element in the development of atherosclerosis and CHD, the activity of PTGS2 may influence the progression of CHD by modulating the inflammatory response. Furthermore, studies have shown^[32]^ that overexpression of PTGS1 and PTGS2 can lead to excessive platelet activation, increasing the risk of thrombosis, causing vasoconstriction, and reducing blood flow,^[31,33]^ thereby impacting the onset and prognosis of CHD in multiple ways. ADRB2 is involved in regulating heart rate and myocardial contractility,^[34]^ making it an important target for CHD treatment. NCOA1 and NCOA2 are 2 nuclear receptor coactivators that participate in the transcriptional coactivation of various signal-activated transcription factors. While direct evidence linking NCOA2 to CHD is limited, its potential role in immune and metabolic regulation^[35]^ may be relevant to the pathogenesis of CHD. NCOA1 is identified as having a substantial impact on cardiac development and function. Abnormal expression or function of NCOA1 may affect cardiac contractility and relaxation, potentially contributing to the development of CHD,^[36]^ and additionally, NCOA1 may influence the development of CHD through its effects on the inflammatory response.^[37]^ RXRA is primarily associated with vitamin A metabolism and signaling, but research has confirmed^[38]^ that under conditions of atherosclerosis, RXRA expression and function may be altered, affecting plaque stability and the inflammatory response. Mutations in the SCN5A gene are mainly linked to arrhythmias, and cardiac electrophysiological abnormalities may indirectly affect cardiac pumping function, impacting myocardial blood supply and oxygenation, and thus influencing the development and prognosis of CHD.^[39,40]^ Research on ESR1 has largely focused on breast cancer, but there is evidence that specific polymorphisms and mutations in the ESR1 gene are connected to the risk factors for CHD.^[41]^ AR intervenes in angiotensin II type 1 receptor-mediated vasoconstriction, oxidative stress, and inflammation, reducing the risk of cardiovascular disease.^[42]^ New evidence hints at a closer link between DPP4 and CHD, with inhibition of DPP4 expression capable of treating CHD through multiple pathways, such as lowering lipid levels^[43–47]^ and reducing monocyte migration to atherosclerotic plaques,^[48]^ making DPP4 a potential candidate target for CHD therapy.^[49]^

To better understand the mechanisms of action of these active ingredients and their clinical significance, we further explored their relationship with enzyme inhibition mechanisms. For example, molecular docking analysis shows that stigmasterol has high binding energy to PTGS2, suggesting that it may exert anti-inflammatory effects by directly inhibiting the activity of this enzyme. PTGS2 (also known as COX-2) is one of the key enzymes in prostaglandin synthesis and plays an important role in inflammatory responses; its overexpression exacerbates inflammation and promotes the progression of atherosclerosis.^[50]^ Therefore, by inhibiting PTGS2 activity, stigmasterol can reduce inflammatory responses and counteract the development of coronary artery disease.^[51]^ In addition, enzyme inhibition not only helps control inflammatory responses but also regulates blood lipid levels, combats oxidative stress, prevents thrombosis, and more.^[52]^ For example, quercetin and luteolin, as flavonoid compounds, can reduce the generation of reactive oxygen species, mitigate oxidative stress damage to the cardiovascular system,^[53]^ and inhibit platelet aggregation to prevent thrombosis,^[54]^ thereby jointly promoting cardiac function improvement and reducing the risk of heart attack. These pharmacological results of enzyme inhibition suggest potential clinical benefits of using BYXD to treat patients with CHD, specifically in the following aspects: First, effectively reducing the release of inflammatory factors by inhibiting key enzyme activity, alleviating the inflammatory state of the coronary artery endothelium;^[55]^ second, the synergistic action of multiple components helps regulate cholesterol levels, reduce low-density lipoprotein oxidation, and delay the process of atherosclerosis;^[56]^ third, antioxidant components such as baicalin can reduce myocardial ischemia–reperfusion injury, protecting myocardial cells from further damage;^[57]^ finally, improving blood flow by inhibiting platelet aggregation and dilating blood vessels, improving myocardial blood supply.^[58]^ These findings provide important scientific evidence for the application of BYXD in the treatment of CHD and demonstrate its broad clinical potential.

Further analysis using KEGG revealed that the mechanism of action of BYXD in treating CHD involves the AGE-RAGE signaling pathway, TNF signaling pathway, HIF-1 signaling pathway, and IL-17 signaling pathway. The AGE-RAGE signaling pathway plays a crucial role in diabetic complications; advanced glycation end products result from non-enzymatic reactions between free reducing sugars and proteins, lipids, or nucleic acids. These AGEs are mainly produced under circumstances of chronic hyperglycemia and aging. Interaction between AGEs and their receptor, RAGE, activates genes, and proteins involved in various signaling pathways. Accumulation of AGEs and upregulation of RAGE expression are associated with various pathological conditions, including coronary atherosclerotic heart disease.^[59]^ Elevated levels of TNF promote atherosclerosis and plaque instability by inducing chemokines/adhesion molecules, impairing vascular motility, and triggering matrix metalloproteinases. Blocking TNF signaling can alleviate inflammation and stabilize atherosclerotic plaques.^[60]^ The HIF-1 signaling pathway serves a crucial function under hypoxic conditions, participating in angiogenesis^[61]^ and regulating the occurrence and development of atherosclerosis.^[62]^ The IL-17 signaling pathway is important in the pathogenesis of CHD. Li et al^[63]^ reported that hyperglycemia can exacerbate coronary atherosclerosis through the tbk1-HIF-1-mediated IL-17/IL-10 signaling pathway.

Drawing from an examination of public databases, we have postulated the possible therapeutic mechanisms of BYXD in addressing CHD. Nonetheless, the effectiveness of BYXD’s active constituents currently lacks the backing of concrete evidence. Furthermore, the pathways through which BYXD may exert its effects on CHD remain unverified by experimental findings. It is recommended that additional inquiry, encompassing both animal and cellular studies, be pursued to validate these mechanisms. As a result, the conclusions drawn from this study are provisional and await confirmation through rigorous empirical research.

This study confirmed through molecular docking that the core components of BYXD exhibit favorable binding activity with the primary therapeutic targets for CHD. This further substantiates that BYXD exerts its effects through modulating multiple BP and signaling pathways, working synergistically to achieve lipid-lowering, anti-inflammatory, antioxidant, antithrombotic, and antiarrhythmic effects, thereby effectively treating CHD. These findings provide strong support for a deeper understanding of the pharmacological actions and clinical applications of BYXD.

### 4.1. Model validation and methodological limitations

#### 4.1.1. Model validation

In this study, we explored the potential mechanisms of BYXD in treating CHD using network pharmacology and molecular docking methods. To validate our model, molecular docking experiments were conducted to assess the binding affinity between key components of BYXD, such as stigmasterol, quercetin, β-sitosterol, luteolin, and baicalein, and primary targets. The results showed that most active ingredients exhibited strong binding potential with core targets, particularly the binding of stigmasterol with PTGS2 and β-sitosterol with NCOA1, further confirming that BYXD achieves its therapeutic effects by regulating multiple BP and signaling pathways.

However, it is important to note that while molecular docking provides an effective method for predicting drug-target interactions, it cannot fully simulate real biological environments. Therefore, these findings need to be validated through in vivo or in vitro experiments in subsequent studies to confirm their actual effects.

#### 4.1.2. Methodological limitations and their impact on results

This study has certain methodological limitations. Firstly, although databases like TCMSP and BATMAN-TCM provide a large amount of information on Chinese herbal medicine components, they do not comprehensively cover all available data, meaning some potentially important components may have been overlooked. Secondly, GO and KEGG analyses rely on existing gene annotation databases, which might not be updated in a timely manner with the latest scientific discoveries, thus limiting the accuracy of functional enrichment analysis.

Moreover, while network pharmacology offers a new perspective for understanding the complex systems of TCM, this approach itself has limitations, such as the difficulty in precisely depicting the specific action pathways of each component and their synergistic effects. Future work needs to address these shortcomings through more in-depth basic research and clinical trials to provide a stronger scientific basis for the mechanisms of BYXD and promote its clinical application.

In summary, although we employed various methods in this study to explore the mechanisms of BYXD in treating CHD, further experimental validation is needed to overcome the limitations of current methods. This will enhance our understanding of the therapeutic effects of BYXD and lay a solid foundation for its clinical application.

## Author contributions

**Conceptualization:** Chuanqian Liu, Zhenzhen Liu.

**Data curation:** Chuanqian Liu, Zhenzhen Liu, Xueting Zhang, Xipeng Yan.

**Funding acquisition:** Xipeng Yan.

**Investigation:** Chuanqian Liu, Zhenzhen Liu.

**Project administration:** Xipeng Yan.

**Resources:** Xueting Zhang, Xipeng Yan.

**Visualization:** Chuanqian Liu, Zhenzhen Liu.

**Writing – original draft:** Chuanqian Liu, Zhenzhen Liu.

**Writing – review & editing:** Chuanqian Liu, Zhenzhen Liu.
